# Visceral leishmaniasis in a peri-urban area of São Paulo associated with Nyssomyia whitmani

**DOI:** 10.11606/s1518-8787.2026060007326

**Published:** 2026-07-24

**Authors:** Amanda Andrade do Rosário, Kleber Agari Campos, Glédson Bandeira Maia, Maria Luiza Bregieiro Abuassi, Gabriela Salvador, Cynthia Piateli, Márcia Dalastra Laurenti, Vânia Lúcia Ribeiro da Matta, Gabriela Araújo Flores, Roberto Mitsuyoshi Hiramoto, Jose Eduardo Tolezano, Fredy Galvis Ovallos

**Affiliations:** I Universidade de São Paulo. Faculdade de Saúde Pública. Departamento de Epidemiologia. São Paulo, SP, Brasil; II Secretaria Municipal da Saúde. Divisão de Vigilância de Zoonoses. São Paulo, SP, Brasil; III Secretaria Municipal da Saúde. Coordenadoria Regional de Saúde Norte - Unidade de Vigilância em Saúde Perus. São Paulo, SP, Brasil; IV Universidade de São Paulo. Faculdade de Medicina. Departamento de Patologia. São Paulo, SP, Brasil; V Universidade Estadual Paulista. Instituto de Biociências. Departamento de Ciências Biológicas e Ambientais. São Vicente, SP, Brasil; VI Instituto Adolfo Lutz. Centro de Parasitologia e Micologia. São Paulo, SP, Brasil

**Keywords:** Infectivity, Bioecology, Secondary Vectors, Leishmaniasis

## Abstract

**OBJECTIVE:**

To investigate an outbreak of canine visceral leishmaniasis and the circulation of *Leishmania* in phlebotomine species in a peri-urban area in the municipality of São Paulo.

**METHODS:**

Twelve dogs were assessed for *Leishmania* infection and sandflies were collected using Shannon and CDC light traps. The presence of the parasite in the captured female sandflies was analyzed by molecular method.

**RESULTS:**

Of the dogs analyzed, four were seropositive both in the rapid serological test and by ELISA for *Le. infantum*. Among the 8,354 phlebotomines captured, *Nyssomyia whitmani* predominated in the area (98.2%) and DNA from *Le. infantum* and *Le. braziliensis* was detected in females of this species, indicating potential participation in the transmission cycle of the visceral leishmaniasis agent.

**CONCLUSION:**

The findings of this study demonstrate the occurrence of an outbreak of canine visceral leishmaniasis in the Perus district, in the municipality of São Paulo, characterized by the presence of dogs infected with *Le. infantum* and potential transmission associated with the phlebotomine *Ny. whitmani*. It also reinforces the importance of entomological surveillance of susceptible vectors and the integration of information in a one health approach, with a view to the early detection of potential transmission foci.

## INTRODUCTION

Considered by the World Health Organization (WHO)^1^ to be among the most important neglected diseases in the world, leishmaniasis is caused by protozoa of the subfamily Leishmaninae, mainly of the genus *Leishmania* (Trypanosomatidae, Kinetoplastida)^2^and is transmitted to animals and humans through the bite of sandflies, insects of the family Psychodidae, subfamily Phlebotominae.

Visceral leishmaniasis (VL) is the most serious form of the disease, with high lethality in humans when left untreated. It affects internal organs such as the liver, spleen and bone marrow, developing months or years after the bite of infected female sandflies, with a worldwide incidence^3^. Although most common in the northeast of the country, VL is expanding geographically, associated with interactions that include environmental and climatic changes, the adaptation of vectors to these new scenarios and the movement of infected dogs to non-endemic areas because of human mobility^4^. All of this implies the possibility of maintaining the parasite cycle in environments occupied by phlebotomine species that are permissible for the parasite to develop.

Although in the state of São Paulo the transmission of VL has been described since the late 1990s with the identification of the presence of the main vector, *Lutzomyia longipalpis* ^5^, in the Greater São Paulo region, cases of canine leishmaniasis dissociated from the presence of this species have been reported since 2005, following the detection of autochthonous cases in the municipality of Embu das Artes^6^. Recently, human and canine cases have also been reported in the municipalities of Itapevi and Cotia, in the Metropolitan Region, and Guarujá, on the state coast^7^. The occurrence of canine cases of VL usually precedes the occurrence of human cases, and confirmation of autochthony is associated with the identification of the presence of vectors^8^. In these locations, the phlebotomine species *Pintomyia fischeri* and *Nyssomyia intermedia* were identified as potential vectors of the parasite^9,10^. The confirmation of species as vectors is associated with different criteria that allow them to be classified as proven or suspected vectors, as recently proposed in an algorithm that classifies species into five categories according to the degree of evidence of their vectorial capacity^11^.

The city of São Paulo has favorable conditions for the establishment of transmission foci, especially in districts located in peri-urban areas, considering the presence of secondary permissive vectors^12^. In this sense, surveillance actions of the agent’s reservoir associated with entomological surveillance activities are fundamental for characterizing new transmission foci, as it provides information that enables the development of more effective prevention and control measures tailored to the characteristics of the area. Therefore, the aim of this study was to characterize the occurrence of the outbreak of canine visceral leishmaniasis (CVL) and to investigate the circulation of *Leishmania* spp. in the species of sandflies present in the area.

## METHODS

### Study Area

The study was carried out in the neighborhood of Perus, located in the northern region of the city of São Paulo, in the district also called Perus (Figure A and B). This neighborhood is characterized by being an urban core isolated from the rest of the city by a green belt. The district is located close to the Bandeirantes highway (SP-348), one of the main access routes to the capital from the Campinas region. It borders the municipality of Caieiras and is considered the second location in the municipality of São Paulo in terms of vegetation cover, made up mainly of ombrophilous forest vegetation and homogeneous forest massifs, corresponding to 75.7% of the territory^13^.

**Figure:**
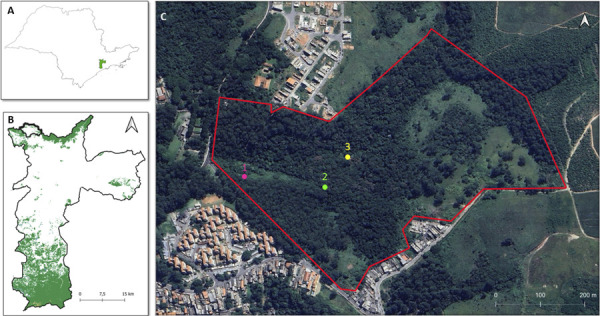
Representative map of the collection site.

Note: (A) location of the city of São Paulo in the state of São Paulo; (B) vegetation cover of the municipality of São Paulo, with emphasis on the location of the Perus neighborhood; (C) area of the property with the three collection points (1) kennel, (2) forest edge and (3) chicken coop.

### Characterization of the Research Site

The property (23°23’56.2”S 46°44’24.6”W) surveyed is located in a forest fragment with a kennel located 200 meters from a housing development. The kennel housed two dogs suspected of having CVL and was built with wood and iron railings. The wooden part of the floor is open to the ground, which allows for the accumulation of organic matter, humidity and shade even during the day. Inside the property there is an uninhabited house that serves as a shelter for domestic animals, mainly chickens, ducks and cats. On the other side of the forest fragment is another housing estate. The average relative humidity and temperature were 81% and 21 ºC respectively.

### Investigation of Suspected CVL Dogs

The presence of dogs with a suspected VL clinic was reported in July 2024 by a veterinarian who treats the dogs on the property. Following the notification, teams from the local Health Surveillance Unit visited the site to collect samples from 12 dogs, detecting four seropositive animals. Subsequently, a team of researchers from the Adolfo Lutz Institute surveyed the site to collect samples and analyze the infection status of the animals. Samples were taken from the four dogs that reacted to the DPP® Canine Visceral Leishmaniasis Rapid Test (RT) Bio-Manguinhos. Venous blood, lymph node, and skin samples were collected for parasitological and molecular diagnosis and attempted isolation in culture. DPP tests, ELISA – LVC Bio-Manguinhos immunoenzymatic assay, PCR of lymph nodes and skin, parasitology and isolation in culture were carried out. At the same time, entomological surveillance activities were started with the aim of identifying the presence of sandflies in the area, identifying the species and investigating the circulation of *Leishmania* spp.

Entomological research activities carried out previously by the São Paulo municipal entomology team in August and September 2024 did not detect the presence of the *Lu. longipalpis* vector, with the identification of 210 males and 205 females, belonging to six species, *Ny. whitmani*, *Pi. fischeri*, *Migonemyia migonei*, *Expapillata firmatoi*, *Psathyromyia pascalei*, and *Brumptomyia nitzulescui*. *Ny. whitmani* accounted for 90% of the specimens caught. These specimens have been deposited in the Collection of Synanthropic Fauna of the Municipality of São Paulo.

### Collection and Identification of Phlebotomines

Considering the previous information obtained which did not detect the presence of the main vector, but with the presence of potential vectors, systematic captures were planned in this study to deepen the entomological investigation. To this end, three capture points were selected, located at the kennel (point 1), at the edge of the forest 50 meters from the kennel (point 2) and in the chicken coop (point 3), approximately 250 meters from the kennel (Figure C). Captures began in October 2024 and, as the presence of the main vector *Lutzomyia longipalpis* was not detected, were extended until March 2025, using CDC, active search and *Shannon* traps. An automatic lighted CDC trap was installed at each point between 6pm and 7am the following day. The *Shannon* trap was installed near the chicken coop from 6pm to 10pm, the same time as the active search for sandflies.

### Phlebotomine Screening and Identification

After capture, the specimens were packaged and transported to the Public Health Entomology Laboratory – Phlebotominae at the University of São Paulo. A sample of the females was dissected to assess the presence of flagellates suggestive of trypanosomatids^14^. Another sample, including specimens captured near the kennel, was sorted and stored in 70% alcohol to be analyzed for the presence of *Leishmania* spp. DNA by molecular method. The other specimens collected were counted and identified morphologically using the Galati identification key^15^.

### Molecular Detection of Leishmania spp. DNA in Sandflies

Of the 3,102 females captured in the kennel, 186 were subjected to molecular analysis by PCR to assess the detection of *Leishmania* spp. DNA. Of these, 30 were analyzed individually and another 156 were grouped into 16 *pools*, with up to 20 females per group. The samples were subjected to DNA extraction^9^and then PCR using primers that amplify the internal transcribed spacer (ITS1) region, to detect the presence of the parasite’s DNA. The thermocycling conditions followed the protocol described by Guimarães et al.^16^. The samples were also tested with specific primers to amplify the hsp70 gene region, according to the methodology proposed by Graça et al.^17^. To characterize the *Leishmania* species*,* the restriction enzymes Hae *III*, *BstUI* and *MbolI* were used using the PCR-RFLP method^18^. The enzyme digestion products were visualized on a 3% agarose gel.

## RESULTS

### Investigation of Leishmania spp. Infection in Dogs

Of the 12 dogs on the property tested, four (33.3%) tested positive for CVL, confirmed by TR DPP and ELISA. Of the four seropositive dogs identified, amastigote forms of *Leishmania* spp. were observed in the skin sample of one of the dogs, and a culture was obtained from the lymph node aspirate of another animal on the property. Molecular analysis detected the presence of *Leishmania* spp. in the skin samples of two animals. The molecular tests made it possible to characterize the presence of *Le. infantum* in the positive samples.

### Phlebotomine Capture

8,354 specimens were collected, of which 57,2% (4.778) were males. Five species were identified belonging to the genera *Nyssomyia*, *Pintomyia*, *Migonemyia*, *Brumptomyia*, and *Psathyromyia* (Table). However, *Ny. whitmani* was the predominant species, accounting for 98.2% of all specimens captured. Other species of epidemiological importance identified were *Pi. fischeri* (1,1%) and *Mg. migonei* (0,7%). The presence of *Lu. longipalpis*, the main vector of the etiologic agent of CVL, was not detected throughout the study period.

**Table:** Distribution of sandflies according to species, sex and capture environment in Perus, between October 2024 and March 2025, in the municipality of São Paulo, SP, Brazil.

Species	Forest edge		Kennel		Chicken coop		Males	Females	%
	Males	Females	Males	Females	Males	Females			
*Nyssomyia whitmani*	248	13	596	298	3,850	3,211	4,694	3,522	98.2
*Pintomyia fischeri*	0	0	11	10	41	30	52	40	1.1
*Migonemyia migonei*	3	0	12	6	17	4	32	10	0.7
*Brumptomyia sp.*	0	0	0	0	0	1	0	1	0
*Psathyromyia pascalei*	0	1	0	0	0	2	0	3	0
Total	251	14	619	314	3,908	3,248	4,778	3,576	100
%	5.3	0.4	13.0	8.8	81.8	90.8			

Regarding the capture points, the chicken coop was the place with the highest frequency of specimens captured (85.6%), followed by the kennel, which accounted for 11.2%. *Ny. whitmani* was the predominant species at all capture points. It should be noted that only *Ny. whitmani*, *Pi. fischeri* and *Mg. migonei* were caught in the kennels.

Of the total number of *Ny. whitmani* females captured (3,522), 153 specimens (4.3%) were dissected to check for the presence of flagellate forms, and 145 (4,2%) were submitted to molecular analysis. In the dissection, suggestive flagellate forms were observed in the region of the stomodeal valve and intestine of two females, but it was not possible to isolate them in culture or characterize them by molecular method. On the other hand, molecular analysis detected the presence of *Leishmania* DNA in three females. It was possible to characterize the *Leishmania* species using the Hae *III* restriction enzyme, and the presence of *Le. infantum* DNA was identified in a female *Ny. whitmani*, and *Le. braziliensis* DNA in another female of this species, both captured at the kennel*.* Additionally, a female *Pi. fischeri* presented *Leishmania* sp. DNA, captured in the chicken coop, but it was not possible to characterize the species.

## DISCUSSION

This study characterized the occurrence of an outbreak of CVL in the Perus district of the city of São Paulo, with the identification of dogs infected with *Le. infantum*. In the Greater São Paulo region, the occurrence of canine cases of VL has been recorded in municipalities close to the city of São Paulo with the occurrence of vector species such as *Pi. fischeri* and *Mg. migonei* ^19^, and human cases reported in the municipalities of Itapevi and Cotia^7^.

During the study period, the presence of *Lu. longipalpis*, a species known to occur in the municipality of Caieiras^20^, was not identified. This result indicates the occurrence of transmission associated with vectors considered secondary in the transmission of the CVL agent. The identification of this outbreak corroborates the process of expansion of CVL into new areas of Greater São Paulo, which has been associated with the introduction of infected dogs^4,9^. Unlike previous studies, in which *Pi. fischeri* was the predominant species in municipalities with feline cases, canine cases, and human cases^9,19,21^, *Ny. whitmani* predominated in the focus studied, representing 98.2% of the phlebotomines captured. This high frequency was observed during all the collections and at all the capture points. This finding is relevant, since vector density is one of the main parameters for assessing the potential of a species to sustain the transmission of a pathogen^22^ and for evaluating potential control strategies^23^, indicating that although other species known for their vector potential, *Pi. fischeri* and *Mg. migonei* were also captured in the area, their densities were inexpressive, 1,1% and 0,7% respectively, indicating *Ny. whitmani* as a potential vector in this location.

A second important element in assessing the participation of a species as a vector is the detection of parasite forms in the females. Although suggestive flagellated forms were observed in two females in the parasitological examination by dissection, it was not possible to isolate and characterize them. On the other hand, in the analysis for parasite DNA, it was possible to detect the presence of *Le. infantum* DNA in one *Ny. whitmani* female. This female contained eggs in her abdomen, indicating possible survival of the parasite during the blood digestion process. *Ny. whitmani* is a species recognized for its anthropophilic habit; therefore, considering the findings of this study, it can be considered a potential vector with level III evidence^11^. Studies still need to be carried out to assess its vectorial competence, to move forward with its incrimination as a vector. The detection of *Le. infantum* DNA in *Ny. whitmani* has also been recorded in other locations in Brazil^24^. Therefore, the evidence obtained so far highlights the need to investigate the potential occurrence of VL foci associated with this species, considering its wide geographical distribution in Brazil, its potential for future geographical expansion^27^ and potential differences in vectorial capacity due to the fact that this taxon is considered a complex of species^28^.

This study also detected the presence of *Le. braziliensis* DNA in a female *Ny. whitmani*. This result suggests the coexistence of potential sources of infection of the cutaneous leishmaniasis agent at the site, drawing attention to the need for entomological surveillance activities in areas close to the study site. A female *Pi. fischeri* was detected with *Leishmania* sp. DNA, but it was not possible to characterize it specifically. This species has a geographical distribution associated with areas where there are outbreaks of CL in the state of São Paulo^29^and is susceptible to *Leishmania braziliensis* ^30^. It is also considered a vector of *Le. infantum* with evidence level IV^11^, lacking only the demonstration of its vectorial competence to be recognized as a proven vector of this agent.

This study highlights the importance of integrating information from different sources on reservoirs, vectors and zoonosis surveillance activities in order to develop a response to emerging or re-emerging problems, such as leishmaniasis, based on a one health approach aimed at the early detection of potential public health problems and the development of evidence-based prevention and control strategies applied at local level. It also highlights the importance of reporting suspected canine cases of the early detection of potential transmission foci and characterization of the vectors involved, considering that anthropogenic changes associated with urbanization, especially in peri-urban areas with the introduction of susceptible people, can constitute a risk for the occurrence of human cases. However, it should be emphasized that the vectorial capacity of the species considered to be permissive vectors is lower than that observed for *Lu. longipalpis*, as previously described in other outbreaks of CVL in Greater São Paulo^9^.

## CONCLUSION

The findings of this study demonstrate the occurrence of an outbreak of CVL in the Perus district, in the municipality of São Paulo, characterized by the presence of dogs infected with *Le. infantum* and potential transmission associated with the phlebotomine *Ny. whitmani*. It also reinforces the importance of entomological surveillance of permissive vectors and the importance of integrating information into a one health approach, with a view to the early detection of potential transmission foci, especially in peri-urban areas subject to disorderly expansion and the introduction of infected animals.

## Data Availability

The research data is available on request from the corresponding author.
