# Supplementary material for: Visceral leishmaniasis in a peri-urban area of São Paulo associated with Nyssomyia whitmani
Source: Rev Saude Publica. 2026 Jul 24;60:e32. doi: 10.11606/s1518-8787.2026060007326 (PMC13398605; doi:10.11606/s1518-8787.2026060007326)
Supplement: Translation

# Translation: Leishmaniose visceral em área periurbana de São Paulo associada a Nyssomyia whitmani

**Authors:** Amanda Andrade do Rosário, Kleber Agari Campos, Glédson Bandeira Maia, Maria Luiza Bregieiro Abuassi, Gabriela Salvador, Cynthia Piateli, Márcia Dalastra Laurenti, Vânia Lúcia Ribeiro da Matta, Gabriela Araújo Flores, Roberto Mitsuyoshi Hiramoto, Jose Eduardo Tolezano, Fredy Galvis Ovallos

**Affiliations:** I Universidade de São Paulo. Faculdade de Saúde Pública. Departamento de Epidemiologia. São Paulo, SP, Brasil; II Secretaria Municipal da Saúde. Divisão de Vigilância de Zoonoses. São Paulo, SP, Brasil; III Secretaria Municipal da Saúde. Coordenadoria Regional de Saúde Norte - Unidade de Vigilância em Saúde Perus. São Paulo, SP, Brasil; IV Universidade de São Paulo. Faculdade de Medicina. Departamento de Patologia. São Paulo, SP, Brasil; V Universidade Estadual Paulista. Instituto de Biociências. Departamento de Ciências Biológicas e Ambientais. São Vicente, SP, Brasil; VI Instituto Adolfo Lutz. Centro de Parasitologia e Micologia. São Paulo, SP, Brasil

**Keywords:** Infectividade, Bioecologia, Vetores Secundários, Leishmaniose

## Abstract

**OBJETIVO:**

Investigar um foco de leishmaniose visceral canina e a circulação de *Leishmania* nas espécies de flebotomíneos em área periurbana do município de São Paulo.

**MÉTODOS:**

Doze cães foram avaliados quanto à infecção por *Leishmania* e foram realizadas coletas de flebotomíneos com armadilhas de Shannon e luminosas do tipo CDC. A presença do parasito nas fêmeas de flebotomíneos capturados foi analisada por método molecular.

**RESULTADOS:**

Dos cães analisados, quatro foram soropositivos tanto no teste sorológico rápido quanto por ELISA para *Le. infantum*. Entre 8.354 flebotomíneos capturados, *Nyssomyia whitmani* predominou na área (98,2%) e foi detectado DNA de *Le. infantum* e *Le. braziliensis* em fêmeas desta espécie, indicando a potencial participação no ciclo de transmissão do agente da leishmaniose visceral.

**CONCLUSÃO:**

Os achados do presente estudo demonstram a ocorrência de um foco de leishmaniose visceral canina no distrito de Perus, município de São Paulo, caracterizado pela presença de cães infectados por *Le. infantum* e potencial transmissão associada ao flebotomíneo *Ny. whitmani*. Também reforça a importância da vigilância entomológica de vetores suscetíveis e da integração de informações em um enfoque de uma só saúde, visando a detecção precoce de potenciais focos de transmissão.

## INTRODUÇÃO

Consideradas pela Organização Mundial da Saúde (OMS)^1^ entre as doenças negligenciadas mais importantes do mundo, as leishmanioses são causadas por protozoários da subfamília Leishmaninae, principalmente do gênero *Leishmania* (Trypanosomatidae, Kinetoplastida)^2^ e é transmitida a animais e humanos por meio da picada de flebotomíneos, insetos da família Psychodidae, subfamília Phlebotominae.

A leishmaniose visceral (LV) é a forma mais grave da doença, com alta letalidade em humanos quando não tratada. Ela afeta órgãos internos como fígado, baço e medula óssea, desenvolvendo-se meses ou anos após a picada de fêmeas de flebotomíneos infectadas, com incidência mundial^3^. Embora mais comum no Nordeste do país, a LV apresenta expansão geográfica associada a interações que incluem mudanças ambientais e climáticas, adaptação dos vetores a esses novos cenários e movimentação de cães infectados para áreas não endêmicas, resultante da mobilidade humana^4^. Tudo isso implica a possibilidade de manutenção do ciclo do parasito em ambientes ocupados por espécies de flebotomíneos permissíveis para o desenvolvimento do parasito.

Embora no estado de São Paulo a transmissão da LV tenha sido descrita desde o fim da década de 1990 com a identificação da presença do principal vetor, *Lutzomyia longipalpis* ^5^, na região da Grande São Paulo , casos de leishmaniose canina dissociada da presença dessa espécie vêm sendo notificados desde 2005, após a detecção de casos autóctones no município de Embu das Artes^6^. Recentemente, casos humanos e caninos também foram notificados nos municípios de Itapevi e Cotia, na Região Metropolitana, e Guarujá, no litoral do estado^7^. A ocorrência de casos caninos de LV geralmente precede a ocorrência de casos humanos, e a confirmação de autoctonia está associada à identificação da presença dos vetores^8^. Nessas localidades as espécies de flebotomíneos *Pintomyia fischeri* e *Nyssomyia intermedia* foram apontadas, respectivamente, como vetores potenciais do parasito^9,10^. A comprovação de espécies como vetores está associada a diferentes critérios que permitem a sua classificação como vetores comprovados e suspeitos, como recentemente proposto em um algoritmo que classifica as espécies em cinco categorias de acordo ao grau de evidências sobre sua capacidade vetorial^11^.

A cidade de São Paulo reúne condições favoráveis para o estabelecimento de focos de transmissão especialmente em distritos situados em áreas periurbanas, considerando a presença de vetores permissivos secundários^12^. Neste sentido, ações de vigilância do reservatório do agente associadas a atividades de vigilância entomológica são fundamentais para a caracterização de novos focos de transmissão, integrando informações que permitam a elaboração de medidas de prevenção e controle mais eficazes de acordo às características do território. Portanto, o presente estudo teve por objetivo caracterizar a ocorrência do foco de leishmaniose visceral canina (LVC) e investigar a circulação de *Leishmania* spp. nas espécies de flebotomíneos presentes na área.

## MÉTODOS

### Área de Estudo

O estudo foi realizado no bairro de Perus, localizado na região norte da cidade de São Paulo, no distrito também chamado de Perus (Figura A e B). Este bairro é caracterizado por ser um núcleo urbano isolado do restante da cidade por um cinturão verde. O bairro encontra-se localizado próximo da rodovia dos Bandeirantes (SP-348), uma das principais rotas de acesso à capital desde a região de Campinas, é limítrofe com o município de Caieiras e é considerada a segunda localidade do município de São Paulo em cobertura vegetal, formada principalmente por vegetação de floresta ombrófila e maciços florestais homogêneos, correspondendo a 75,7% do território^13^.

**Figura:** Mapa representativo do local de coleta.

Nota: (A) localização da cidade de São Paulo no estado de São Paulo; (B) cobertura vegetal do município de São Paulo, com destaque para a localização do bairro Perus; (C) área da propriedade com os três pontos de coleta (1) canil, (2) borda de mata e (3) galinheiro.

### Caracterização do Local da Pesquisa

A propriedade (23°23’56.2”S 46°44’24.6”W) pesquisada localiza-se em um fragmento de mata com presença de um canil localizado a 200 metros de um conjunto habitacional. O canil abrigava dois cães com suspeita de LVC, construído com madeira e grades de ferro. A parte de madeira do piso, é vazada para o solo, o que permite o acúmulo de matéria orgânica, umidade e sombra mesmo durante o dia. No interior da propriedade há uma casa não habitada que serve de abrigo de animais domésticos, principalmente galinhas, patos e gatos. Do outro lado do fragmento florestal é encontrado outro conjunto habitacional. A média de umidade relativa e temperatura, foi respectivamente 81% e 21ºC.

### Investigação de Cães Suspeitos LVC

A presença de cães com clínica suspeita de LV foi notificada em julho de 2024 por uma médica veterinária que atende os cães da propriedade. Após a notificação, equipes da Unidade de Vigilância em Saúde da localidade realizaram visita ao local para coleta de amostras de 12 cães, detectando quatro animais soropositivos. Posteriormente, uma equipe de pesquisadores do Instituto Adolfo Lutz realizou a pesquisa no foco para coleta de amostras e análise do status de infecção dos animais. Foi realizada a coleta de amostras dos quatro cães reagentes no Teste Rápido (TR) DPP® Leishmaniose Visceral Canina Bio-Manguinhos. Foram coletadas amostras de sangue venoso, linfonodo e pele para diagnóstico parasitológico e molecular e tentativa de isolamento em cultura. Foram realizados testes DPP, ensaio imunoenzimatico ELISA – LVC Bio-Manguinhos, PCR de linfonodo e pele, parasitológico e isolamento em cultura. Concomitantemente, foram iniciadas atividades de vigilância entomológica com o objetivo de identificar a presença de flebotomíneos na área, identificação das espécies e investigação da circulação de *Leishmania* spp.

Atividades de pesquisa entomológica realizadas previamente pela equipe de entomologia do município de São Paulo em agosto e setembro de 2024, não detectaram a presença do vetor *Lu. longipalpis*, com identificação de 210 machos e 205 fêmeas, pertencentes a seis espécies, *Ny. whitmani*, *Pi. fischeri*, *Migonemyia migonei*, *Expapillata firmatoi*, *Psathyromyia pascalei* e *Brumptomyia nitzulescui*. *Ny. whitmani* representou 90% dos espécimes capturados. Espécimes estes depositados na Coleção de Fauna Sinantrópica do Município de São Paulo.

### Coleta e Identificação de Flebotomíneos

Considerando as informações prévias obtidas que não detectaram a presença do principal vetor, porém com presença de potenciais vetores, foram planejadas capturas sistemáticas no presente estudo para aprofundar a investigação entomológica. Para isso, foram selecionados três pontos de captura, localizados no canil (ponto 1), na borda da mata distante a 50 metros do canil (ponto 2) e no galinheiro (ponto 3), distante aproximadamente a 250 metros do canil (Figura C). As capturas foram iniciadas em outubro de 2024 e, como não foi detectada a presença do principal vetor *Lutzomyia longipalpis*, se estenderam até março de 2025, usando armadilhas CDC, busca ativa e *Shannon*. Em cada ponto foi instalada uma armadilha automática luminosa do tipo CDC instaladas entre as 18h e às 07h do dia seguinte. A armadilha de *Shannon* foi instalada próxima ao galinheiro das 18h às 22h, mesmo horário em que ocorreu a busca ativa por flebotomíneos.

### Triagem e Identificação de Flebotomíneos

Após a captura, os espécimes foram acondicionados e transportados até o Laboratório de Entomologia em Saúde Pública – Phlebotominae da Universidade de São Paulo. Uma amostra das fêmeas foi dissecada para avaliar a presença de flagelados sugestivos de tripanosomatídeos^14^. Outra amostra incluindo os espécimes capturados próximos ao canil foram triados, acondicionados em álcool 70% para análise da presença de DNA de *Leishmania* spp. por método molecular. Os demais espécimes coletados foram contabilizados e identificados morfologicamente seguindo a chave de identificação de Galati^15^.

### Detecção Molecular de DNA de Leishmania spp. nos Flebotomíneos

Das 3.102 fêmeas capturadas no canil, 186 foram submetidas a análise molecular por PCR para avaliar a detecção de DNA de *Leishmania* spp. Destas, 30 foram analisadas individualmente e outras 156 agrupadas em 16 *pools*, com até 20 fêmeas por grupo. As amostras foram submetidas ao procedimento de extração de DNA^9^ e posteriormente, à PCR utilizando *primers* que amplificam a região do espaçador transcrito interno (ITS1), com o objetivo de detectar a presença do DNA do parasito. As condições de termociclagem seguiram o protocolo descrito por Guimarães et al.^16^ . As amostras também foram testadas com primers específicos para amplificação da região do gene *hsp*70, conforme metodologia proposta por Graça et al.^17^ . Para caracterização da espécie de *Leishmania,* foram utilizadas as enzimas de restrição Hae *III*, Bst*UI* e Mbo*lI* pelo método de PCR-RFLP^18^. Os produtos da digestão enzimática foram visualizados em gel de agarose a 3%.

## RESULTADOS

### Investigação de Infecção por Leishmania spp. nos Cães

Dos 12 cães da propriedade testados, quatro (33,3%) tiveram resultados positivos para LVC confirmados pelo TR DPP e por ELISA. Dos quatro cães soropositivos identificados, foram observadas formas amastigotas de *Leishmania* spp. na amostra de pele de um dos cães, e foi obtida cultura a partir de aspirado de linfonodo de um outro animal da propriedade. Na análise molecular, foi detectada a presença de *Leishmania* spp. nas amostras de pele de dois animais. Os testes moleculares permitiram caracterizar a presença de *Le. infantum* nas amostras positivas.

### Captura de Flebotomíneos

Foram coletados 8.354 espécimes, dos quais 57,2% (4.778) eram machos. Foram identificadas cinco espécies pertencentes aos gêneros *Nyssomyia*, *Pintomyia*, *Migonemyia*, *Brumptomyia* e *Psathyromyia* (Tabela). No entanto, *Ny. whitmani* foi a espécie predominante, correspondendo a 98,2% do total de espécimes capturados. Outras espécies de importância epidemiológica identificadas foram *Pi. fischeri* (1,1%) e *Mg. migonei* (0,7%). Durante todo o período do estudo não foi detectada a presença de *Lu. longipalpis*, principal vetor do agente etiológico da LVC.

**Tabela:** Distribuição de flebotomíneos segundo espécie, sexo e ambiente de captura em Perus, entre os meses de outubro de 2024 e março de 2025, no município de São Paulo, SP, Brasil.

Espécie	Borda de mata		Canil		Galinheiro		Machos	Fêmeas	%
	Machos	Fêmeas	Machos	Fêmeas	Machos	Fêmeas			
*Nyssomyia whitmani*	248	13	596	298	3.850	3.211	4.694	3.522	98,2
*Pintomyia fischeri*	0	0	11	10	41	30	52	40	1,1
*Migonemyia migonei*	3	0	12	6	17	4	32	10	0,7
*Brumptomyia sp.*	0	0	0	0	0	1	0	1	0
*Psathyromyia pascalei*	0	1	0	0	0	2	0	3	0
Total	251	14	619	314	3.908	3.248	4.778	3.576	100
%	5,3	0,4	13,0	8,8	81,8	90,8			

Em relação aos pontos de captura, o galinheiro foi o lugar com maior frequência de espécimes capturados (85,6%), seguido pelo canil que representou 11,2%. Em todos os pontos de captura, *Ny. whitmani* foi a espécie predominante. Ressalta-se que nas capturas no canil, somente *Ny. whitmani*, *Pi. fischeri* e *Mg. migonei* foram capturadas.

Do total de fêmeas de *Ny. whitmani* capturadas (3.522), foram dissecadas 153 espécimes (4,3%) para verificação da presença de formas flageladas, e 145 (4,2%) foram submetidas à análise molecular. Na dissecção, foram observadas formas sugestivas de flagelados na região da válvula estomodeal e intestino de duas fêmeas, porém, não foi possível seu isolamento em cultura, nem sua caracterização por método molecular. Por outro lado, na análise molecular foi detectada a presença de DNA de *Leishmania* em três fêmeas. Foi possível a caracterização da espécie de *Leishmania*, com a utilização da enzima de restrição Hae *III*, sendo identificada a presença de DNA de *Le. infantum* em uma fêmea de *Ny. whitmani*, e de DNA de *Le. braziliensis* em outra fêmea dessa espécie, ambas capturadas no canil*.* Adicionalmente uma fêmea de *Pi. fischeri* apresentou DNA de *Leishmania* sp., capturada no galinheiro, porém não foi possível a caracterização da espécie.

## DISCUSSÃO

No presente estudo foi caracterizada a ocorrência de um foco de LVC no distrito de Perus da cidade de São Paulo, com identificação de cães infectados com *Le. infantum*. Na região da Grande São Paulo, a ocorrência de casos caninos de LV tem sido registrada em municípios próximos à cidade de São Paulo com ocorrência de espécies de vetores como *Pi. fischeri* e *Mg. migonei* ^19^, e casos humanos notificados nos municípios de Itapevi e Cotia^7^.

Durante o período de estudo não foi identificada a presença de *Lu. longipalpis*, espécie com ocorrência conhecida no município de Caieiras^20^. Este resultado indica a ocorrência de transmissão associada a vetores considerados secundários na transmissão do agente da LVC. A identificação do presente foco, corrobora o processo de expansão da LVC em novas áreas da Grande São Paulo, o qual tem sido associado à introdução de cães infectados^4,9^. Diferente de estudos prévios, nos quais *Pi. fischeri* foi a espécie predominante nos municípios com ocorrência de caso felino, casos caninos e humanos^9,19,21^, no foco estudado houve predominância de *Ny. whitmani*, representando 98,2% dos flebotomíneos capturados. Esta elevada frequência foi observada durante todas as coletas realizadas e em todos os pontos de captura. Este achado é de relevância, uma vez que a densidade de vetor é um dos principais parâmetros para a avaliação do potencial de uma espécie para sustentar a transmissão de um patógeno^22^ e para avaliação de potenciais estratégias de controle^23^, indicando que embora outras espécies conhecidas pelo seu potencial vetorial, *Pi. fischeri* e *Mg. migonei* também foram capturadas na área, as suas densidades foram inexpressivas, respectivamente 1,1% e 0,7%, indicando *Ny. whitmani* como um vetor potencial nessa localidade.

Um segundo elemento importante na avaliação da participação de uma espécie como vetora é a detecção de formas do parasito nas fêmeas. Embora formas flageladas sugestivas tenham sido observadas em duas fêmeas no exame parasitológico por dissecção, não foi possível seu isolamento e caracterização. Por outro lado, na análise para detecção de DNA do parasito, foi possível detectar a presença do DNA de *Le. infantum* em uma fêmea de *Ny. whitmani*. Esta fêmea continha ovos no abdômen, indicando possível sobrevivência do parasito durante o processo de digestão do sangue. *Ny. whitmani* é uma espécie reconhecida por seu hábito antropofílico, portanto, considerando os achados do presente estudo, pode ser considerada um potencial vetor com grau de evidência nível III^11^. Estudos ainda devem ser realizados para avaliar a sua competência vetorial, para avançar na incriminação como vetor. A detecção de DNA de *Le. infantum* em *Ny. whitmani* também tem sido registrada em outras localidades do Brasil^24^. Portanto, as evidências obtidas até o momento trazem à tona a necessidade de investigar a potencial ocorrência de focos de LV associados a esta espécie, considerando sua ampla distribuição geográfica no Brasil, seu potencial de expansão geográfica futura^27^ e potenciais diferenças na capacidade vetorial pelo fato deste táxon ser considerado um complexo de espécies^28^.

No presente estudo também foi detectada a presença de DNA de *Le. braziliensis* em uma fêmea de *Ny. whitmani*. Este resultado sugere a coexistência no foco de potenciais fontes de infecção do agente da leishmaniose cutânea, chamando a atenção para a necessidade de atividades de vigilância entomológica nas áreas próximas ao local de estudo. Uma fêmea de *Pi. fischeri* foi detectada com DNA de *Leishmania* sp., todavia não foi possível sua caracterização específica. Esta espécie apresenta distribuição geográfica associada a áreas de ocorrência de focos de LC no estado de São Paulo^29^e é susceptível ao desenvolvimento de *Leishmania braziliensis* ^30^. Também é considerada um vetor de *Le. infantum* com grau de evidência IV^11^, faltando somente a demonstração da sua competência vetorial para ser reconhecida como vetora comprovada desse agente.

O presente trabalho evidencia a importância da integração de informações de diferentes fontes sobre reservatórios, vetores e atividades de vigilância de zoonoses para o desenvolvimento de resposta a problemas emergentes ou reemergentes, como no caso das leishmanioses, fundamentados no enfoque de uma só saúde (*one health*) visando a detecção precoce de potenciais problemas de saúde pública e a elaboração de estratégias de prevenção e controle baseados em evidência aplicadas a nível local. Também ressalta a importância do papel da notificação de casos caninos suspeitos para a detecção precoce de potenciais focos de transmissão e caracterização dos vetores envolvidos, considerando que mudanças antrópicas associadas à urbanização, principalmente em áreas periurbanas com introdução de pessoas suscetíveis podem constituir um risco para a ocorrência de casos humanos. No entanto, deve ser ressaltado que a capacidade vetorial das espécies consideradas vetores permissivos é menor do que observada para *Lu. longipalpis*, como previamente descrito em outros focos de LVC da Grande São Paulo^9^.

## CONCLUSÃO

Os achados deste estudo demonstram a ocorrência de um foco de LVC no distrito de Perus, município de São Paulo, caracterizado pela presença de cães infectados por *Le. infantum* e potencial transmissão associada ao flebotomíneo *Ny. whitmani*. Também reforça a importância da vigilância entomológica de vetores permissivos e a importância da integração de informações em um enfoque de uma só saúde, visando a detecção precoce de potenciais focos de transmissão especialmente em áreas periurbanas sujeitas à expansão desordenada e à introdução de animais infectados.

## Data Availability

Os dados da pesquisa estão disponíveis mediante solicitação ao autor correspondente.
